# GRANDPARENTAL CHILDCARE AND ITS RELATIONSHIP WITH SOCIOECONOMIC INEQUALITIES AND HEALTH: EVIDENCE FROM ENGLAND

**DOI:** 10.1093/geroni/igz038.1046

**Published:** 2019-11-08

**Authors:** Giorgio Di Gessa, Karen Glaser

**Affiliations:** King’s College London, London, United Kingdom

## Abstract

It is well recognised that grandparents play a vital economic and social role in providing grandchild care to families. In the UK, about two million grandparents provide care to their grandchildren for at least ten hours per week. However, to date, little is known about the nature and extent of care they provide and whether, and how, this relates to socio-economic and health inequalities. In this paper, we use wave 8 of the nationally representative English Longitudinal Study of Ageing. Using latent class analysis, we developed profiles of grandparent childcare combining newly collected information on the activities grandparents do with or for their grandchildren, as well as on the extent of (periodicity, frequency, and intensity), and motivations for such care. Using regressions, we then examined the extent to which grandparents’ involvement in childcare is patterned according to current and lifetime socio-economic characteristics and whether such inequalities are an important modifier of the relationship between grandparenting and mental and physical health. Preliminary results suggest that more advantaged grandparents are the ones undertaking less arduous caring tasks and for shorter periods whereas those looking after grandchildren more intensively and for financial reasons are more likely to be of less advantageous backgrounds (not married, low-educated, not homeowners, and poorer). Moreover, undertaking more frequent and challenging grandchild care activities seems to be associated with poorer health but only among grandparents in the more disadvantaged groups. Our results contribute to better understand the nature of grandparental childcare and its effect on health.

